# Parental Migration, Self-Efficacy and Cigarette Smoking among Rural Adolescents in South China

**DOI:** 10.1371/journal.pone.0057569

**Published:** 2013-03-08

**Authors:** Yang Gao, Liping Li, Emily Y. Y. Chan, Joseph Lau, Sian M. Griffiths

**Affiliations:** 1 The J C School of Public Health and Primary Care, The Chinese University of Hong Kong, Hong Kong, China; 2 Injury Prevention Research Center, Medical College of Shantou University, Shantou, China; Old Dominion University, United States of America

## Abstract

**Objectives:**

Millions of children and adolescents in rural China are left behind as their parents move away for work. Little is known about the impact of parental migration on their smoking and self-efficacy. This study explores the associations among parental migration, self-efficacy and smoking.

**Methods:**

A cross-sectional study was conducted among middle school students in Liangying Township, Guangdong, China. Socio-demographic and parental migration characteristics, as well as adolescent past 30-day smoking and self-efficacy level were collected using a self-administered questionnaire. Univariate and multivariate analyses were performed to estimate the risk of parental migration features for smoking and self-efficacy. Hierarchical regression was fitted to examine the relationship among parental migration, self-efficacy and smoking.

**Results:**

2609 students (93.4%) participated into the study, 44% of who were with parents who had ever been or were currently migrating. Smoking prevalence was 9.7% in boys and 0.9% in girls. Paternal migration was protective for adolescent smoking, whilst maternal migration increased the risk. Both paternal and maternal migration had adverse effects on self-efficacy, a strong influencing factor for smoking. No significant relationship was found between other migration features and smoking and self-efficacy. The smoking risk of maternal migration was partly mediated by self-efficacy. There were no differences between boys and girls.

**Conclusions:**

Our findings suggest that adolescents whose mothers migrate from home to work elsewhere are at elevated risk for smoking. Improving self-efficacy may be an effective means to keep adolescents away from smoking, especially for those with maternal migration.

## Introduction

The current tobacco epidemic is the leading preventable cause of death. It is estimated that globally one out of 10 adult deaths are contributed from cigarette smoking and nearly 6 million people per year die from either tobacco use or exposure to tobacco smoke [1]. China has the world’s largest population of smokers and one out of three cigarettes are consumed by Chinese [2]. Near 60% of Chinese males are current smokers, whilst only 4% of females currently smoke [1], [2]. In terms of children and adolescents, the current smoking prevalence reported by different studies varied from 2.7%–17.1% for boys and 0.8%–6.6% for girls [1]–[8]. Although a slight decrease in smoking has been observed among adult males in the past decade, child and adolescent smoking has been continuously increasing, especially among rural boys [2].

Self-efficacy refers to the confidence in one’s ability to behave in such a way as to produce a desirable outcome [9]. It makes a difference in how people feel, think, and act. Self-efficacy as a strong cognitive predictor of smoking has been thoroughly studied. It has been well documented that a low sense of self-efficacy is positively associated with smoking, whilst high self-efficacy allows one to be more likely to quit smoking in the future [10].

Parental and familial factors also play an important role in child and adolescent smoking, as the family unit is the primary source of transmission of basic social, cultural, genetic and biological factors that may underlie individual differences in smoking [11], [12]. In mainland China, around 17.4 million rural children and adolescents are living with one or both of their parents’ absence, as they have moved out from their hometown for work [13]. However, little is known about the impact of parental migration on offspring smoking. Our previous study in rural south China observed that adolescents (aged 10–18 years) with parental migration were at elevated risks for several health related behaviours including cigarette smoking [8]. Past 30-day smoking prevalence in adolescents with parental migration was higher than those without parental migration (12.4% vs. 8.4%), similar to findings from a study in Hunan Province [8], [14]. That study investigated 683 primary and secondary school students in rural areas and revealed an almost double prevalence of past 30-day smoking in students with parental migration in comparison to those without parental migration (9.5% vs. 5.9%) [14]. In this study we explored whether there was any difference between paternal and maternal migration in their associations with adolescent smoking, whether the associations were similar among boys and girls, and whether any features of migration contributed to the associations. In addition, we also investigated adolescent self-efficacy and examined the associations among parental migration, adolescent self-efficacy and smoking.

## Methods

### Participants

This study was conducted in two junior secondary schools in Liangying Township, Shantou City, Guangdong Province, China in November, 2009. Liangying covers an area of 72.4 square kilometres and has a population of about 200 thousand living in 70 villages [15]. It is classified as Class 2 out of four classes of rural China (from very affluent to very poor), indicating that the living standard in Liangying is relatively high. In 2009, the annual income per capita in Liangying was 5,210 RMB, higher than the national average for rural areas (4,140 RMB) [15], [16]. There are ten junior secondary schools in Liangying, of which two schools sharing similar characteristics (e.g. school area, school level, facilities, number of total students, and school policies on student smoking) were selected and recruited into the study.

### Ethics Statement

Ethics approval was obtained from the Ethics Committees at the Chinese University of Hong Kong (CRE-2009.507-T). All students (Grades 7–9) were invited to participate in the study and informed written consent was obtained from their parents in advance.

### Data Collection and Measures

A self-administered questionnaire was applied to collect data. The participants were asked to complete the questionnaire in their classroom. Investigators introduced the study purposes and the instruction on how to fill in the questionnaire in advance, and then responded to questions raised up by the participants. The following information was obtained:

#### Socio-demographic characteristics

Age, gender, ethnicity, school, grade, family income, education of parents and guardian (the person who is in charge of the student’s life and study) were reported by the participants. In addition, they were also asked about their perception of living standard of their family to surrogate family income as our previous study showed that most students did not know their family income [8].

#### Parental migration characteristics

Paternal and maternal migration status was collected by asking two questions: “Has your father ever migrated outside for work for six months or above in a row?” and “Has your mother ever migrated outside for work for six months or above in a row?” [8]. Students gave their choice on three options, namely “Never”, “Yes, he/she has migrated outside for work, but he/she is now at home”, and “Yes, he/she has migrated outside for work and now is still working outside”. For those choosing “Yes” (either of the latter two options), information of cumulative migration time, frequency of returning home and frequency of contact with child during migration was further collected, which was then regrouped according to their distribution and analysed as dichotomous variables. In order to indicate different combinations of paternal and maternal migration, namely, no parent migrating, father migrating only, mother migrating only, and both migrating, a new variable *‘type of parental migration’* was created after combining ever and current migration status, as their associations with smoking and self-efficacy were similar.

#### Past 30-day smoking

Past 30-day smoking was defined as “ever smoked in the past 30 days” [8]. Two questions were asked to the students: “In the past 30 days, how many days did you smoke?” and “In the past 30 days, how many cigarettes did you smoke in a usual day when you smoked?” Options for the first question consisted of “0 day”, “1–2 days”, …, “30 days” and those for the second question included “never smoked’, “less than 1 cigarette”, “1 cigarette”, …, “more than 20 cigarettes” [8]. Students reporting “0 day” and “never smoked” (those choosing the first option for each question) were classified as “did not smoke in the past 30 days”. Students reporting that they smoked at least one day in the past 30 days and that they smoked even less than 1 cigarette (those choosing the second option or afterwards for the both questions) were classified as “ever smoked in the past 30 days”. Those with inconsistent answers (<1%) were treated as missing data.

#### Self-efficacy of smoking

Self-efficacy was measured by the Chinese version of the Smoking Self-Efficacy Questionnaire (SEQ-12), which was translated from the original English SEQ-12 and proved to be a valid and reliable instrument for Chinese [17]. It consists of 12 items measuring two dimensions: internal stimuli (intrapersonal and physiological factors) and external stimuli (social factors). The responses range on the 5-point Likert scale from 1 (strongly disagree) to 5 (strongly agree), with a higher score indicating higher self-efficacy. Cronbach’s alpha for the entire scale was 0.957, higher than those of the two subscales (0.920 for the internal subscale and 0.917 for the external subscale respectively). Mean for the scores (ranged from 1–5) was calculated for each participant, whom was then categorised into “low self-efficacy” or “middle and high self-efficacy” group according to his/her mean of being below or above the cut-off point of the 25^th^ percentile (3.643 for boys and 4.462 for girls respectively).

### Analysis

SPSS for windows (16.0) was used to analyse the data. Mean and standard deviation (SD) and percentage were used to describe the distribution of all variables. Chi-square was performed to compare between-group difference in past 30-day smoking and low self-efficacy. Univariate and multivariate logistic regression were fitted to estimate the risks of migration related variables for smoking and self-efficacy before and after adjustment for socio-demographic factors, including school, grade, ethnicity, gender and education level of the guardian and perceived living standard (as a surrogate of family income). Odds Ratio (OR) and 95% confidence intervals (95% CI) were then derived. In summary, a hierarchical logistic regression was performed to test the relationship among parental migration, self-efficacy and smoking by firstly regressing smoking with migration variables with controlling for the socio-demographic factors, and then entering self-efficacy into the model. Migration variables were stepwisely selected using P<0.20 and P<0.25 as entry and removal criteria. Each of them was separately entered the model in order to avoid multicollinearity problems [18]. The risks for smoking and self-efficacy were estimated in boys and girls separately. However, the socio-demographic factors were not adjusted for smoking among girls (including multivariate and hierarchical logistic regression for smoking) because of the small number of female smoking cases (n = 8).

## Results

A total of 2609 students (93.4%) agreed to participate into the study and 2558 (91.6%) with valid data were analysed. The participants aged from 11–19 years old, with the mean and SD being 13.8 and 1.1 years respectively. Boys (55.0%) were more than girls. Table 1 shows the socio-demographic and parental migration characteristics of the participants. Almost all students’ ethnicity was Han (99.1%), >90% of students reported parents as their guardians, half of whom had an education level of primary school or below, and around 60% of students rated their families in the lower half of perceived living standard. In terms of parental migration characteristics, more fathers than mothers had ever migrated or were currently migrating (41.3% vs. 14.5%). More migrant mothers tended to have a shorter cumulative migration time (<3 years) and return home more often (≥once per month) compared to migrant fathers, whilst the frequency of contact with child was similarly distributed between the two. About 44% of the participants were with single or both parents migrating, specifically, 29.9% were with migrant father only, 3.4% with migrant mother only and 10.8% with both migrating. There were no significant gender differences in migration indicators, except more migrant mothers and less frequency of returning home of migrant mothers were found in boys.

**Table 1:** Socio-demographic and parental migration characteristics of the participants.

	All (%)	Boys (%)	Girls (%)
Socio-demographic indicators	(n = 2558)	(n = 1407)	(n = 1151)
Grade			
7	44.5	42.6	46.9
8	33.0	34.7	30.8
9	22.5	22.7	22.2
* Age* a *(years, Mean, SD)*	*13.8 (1.14)*	*13.9 (1.15)*	*13.6 (1.11)*
Ethnicity			
Han	99.1	99.3	98.9
Others	0.9	0.7	1.1
Guardian			
Father	56.7	61.8*	50.7*
Mother	35.9	31.2*	41.4*
Grandparent	3.0	2.1*	4.1*
Others	4.3	4.8*	3.8*
Education level of guardian			
Primary and low	44.9	41.6*	48.9*
Junior Secondary	41.6	42.6*	40.3*
Senior Secondary and above	13.5	15.8*	10.8*
Perceived living standard			
Low (≤50%)	62.9	66.1*	59.1*
High (>50%)	37.1	33.9*	40.9*
**Parental migration status**	**(n = 2558)**	**(n = 1407)**	**(n = 1151)**
Paternal migration status			
No	58.8	58.8	58.8
Yes, ever	30.2	29.8	30.7
Yes, currently	11.0	11.4	10.6
Maternal migration status			
No	85.5	82.9*	88.6*
Yes, ever	10.6	12.2*	8.6*
Yes, currently	3.9	4.9*	2.8*
Type of parental migration^b^			
No parent migrating	56.0	54.6*	57.6*
Father migrating only	29.9	28.6*	31.4*
Mother migrating only	3.4	4.2*	2.3*
Both migrating	10.8	12.6*	8.7*
**Paternal migration characteristics** c	**(n = 909)**	**(n = 496)**	**(n = 413)**
Cumulative migration time			
<3 years	57.4	57.1	57.9
≥3 years	42.6	42.9	42.1
Frequency of returning home			
≥ Once per month	39.8	41.6	37.8
< Once per month	60.1	58.4	62.2
Frequency of contact with child			
≥ Once per month	66.0	65.1	67.1
< Once per month	34.0	34.9	32.9
**Maternal migration characteristics** d	**(n = 311)**	**(n = 197)**	**(n = 114)**
Cumulative migration time			
<3 years	62.1	64.5	57.9
≥3 years	37.9	35.5	42.1
Frequency of returning home			
≥ Once per month	62.4	67.2*	54.1*
< Once per month	37.6	32.8*	45.9*
Frequency of contact with child			
≥ Once per month	69.2	70.7	66.3
< Once per month	30.8	29.3	33.7

a The range of age was 11–19 years old.

b Ever and current migration status were combined when creating the variable of ‘type of parental migration’.

c Among participants with paternal migration only.

d Among participants with maternal migration only.

* Significant difference (P<0.05) between boys and girls by chi-square test.

The overall prevalence of past 30-day smoking was 5.6% for the entire participants, 9.7% of boys had smoked in the past 30 days, but only 0.9% of girls had smoked in that period (P<0.001). The mean for the SEQ-12 scores was significantly lower among boys than girls (4.21 vs. 4.54, P<0.001) and among smokers than non-smokers (4.77 vs. 5.11, P<0.001). Table 2 presents the percentages and risk estimations of smoking and low self-efficacy by parental migration among boys. Paternal migration seemed to be protective for adolescent smoking, whilst maternal migration was likely to increase the risk when considered individually. Both paternal and maternal migration increased the likelihood of low self-efficacy. The risks of ever and current migration status were similar except a higher OR for low self-efficacy was obtained in those with mother currently migrating after controlling for socio-demographic factors. After combining paternal and maternal migration together, boys with mother migrating only had the highest risk for both smoking and low self-efficacy, followed by those with both parents migrating. Boys with father migrating only had a lower risk for smoking but an increased risk for self-efficacy. No other migration characteristics, including cumulative migration time, frequency of returning home and frequency of contact with child was significantly associated with smoking and self-efficacy. The direction and magnitude of the relationship between parental migration and adolescent self-efficacy in girls seemed to be similar to those in boys, except lower risks of current maternal migration and mother migrating only were found in girls (Table 3). In addition, mother less frequently contacting child during migration seemed to increase the likelihood of low self-efficacy in girls.

**Table 2:** Percentages and risk estimations of past 30-day smoking and low self-efficacy^a^ by parental migration among boys.

		Past 30-day smoking											Low self-efficacy^a^											
		n	%		OR_unadj._	*(95% CI)*		P value	OR_adj._	*(95% CI)*		P value		n	%	OR_unadj._	*(95% CI)*		P value		OR_adj._	*(95% CI)*		P value
**Parental migration status** b																								
Paternal migration status																								
No *(reference)*		71	10.5		1.00				1.00					162	23.0	1.00					1.00			
Yes, ever		25	7.6		0.70	*(0.44, 1.13)*		0.141	0.69	*(0.41, 1.18)*		0.177		107	29.9	1.43	*(1.07, 1.90)*		0.015		1.40	*(1.01, 1.94)*		0.045
Yes, currently		11	8.9		0.83	*(0.43, 1.61)*		0.579	0.70	*(0.32, 1.55)*		0.378		37	27.2	1.25	*(0.83, 1.90)*		0.293		1.20	*(0.73, 1.96)*		0.474
Maternal migration status																								
No *(reference)*		79	8.6		1.00				1.00					205	21.3	1.00					1.00			
Yes, ever		19	15.0		1.87	*(1.09, 3.20)*		0.023	1.79	*(0.96, 3.36)*		0.068		60	42.3	2.70	*(1.87, 3.90)*		<0.001		2.14	*(1.40, 3.25)*		<0.001
Yes, currently		9	18.4		2.39	*(1.12, 5.10)*		0.024	1.63	*(0.65, 4.10)*		0.299		32	56.1	4.73	*(2.74, 8.16)*		<0.001		3.77	*(2.07, 6.89)*		<0.001
Type of parental migration																								
No parent migrating *(reference)*		56	9.5		1.00				1.00					122	19.9	1.00					1.00			
Father migrating only		18	6.0		0.60	*(0.35, 1.05)*		0.073	0.61	*(0.34, 1.10)*		0.098		74	23.1	1.21	*(0.87, 1.68)*		0.252		1.27	*(0.88, 1.82)*		0.204
Mother migrating only		11	25.0		3.17	*(1.52, 6.61)*		0.002	3.03	*(1.35, 6.83)*		0.007		28	59.6	5.93	*(3.21, 10.97)*		<0.001		4.66	*(2.32, 9.36)*		<0.001
Both migrating		15	12.2		1.32	*(0.72, 2.42)*		0.370	1.27	*(0.65, 2.47)*		0.484		60	42.3	2.95	*(2.00, 4.34)*		<0.001		2.43	*(1.57, 3.76)*		<0.001
**Paternal migration characteristics** c																								
Cumulative migration time																								
<3 years *(reference)*		22		8.4	1.00				1.00					81	28.7	1.00				1.00				
≥3 years		17		8.9	1.06	*(0.55, 2.05)*		0.864	1.65	*(0.78, 3.49)*		0.191		66	31.1	1.12	*(0.76, 1.65)*		0.562	1.31		*(0.84, 2.06)*		0.235
Frequency of returning home																								
≥ Once per month *(reference)*		19		10.4	1.00				1.00					66	32.5	1.00				1.00				
< Once per month		18		6.8	0.63	*(0.32, 1.23)*		0.172	0.74	*(0.54, 1.60)*		0.440		79	27.9	0.80	*(0.54, 1.19)*		0.275	0.83		*(0.53, 1.31)*		0.428
Frequency of contact with child																								
≥ Once per month *(reference)*		25		9.0	1.00				1.00					88	28.9	1.00				1.00				
< Once per month		12		7.8	0.85	*(0.42, 1.75)*		0.662	0.92	*(0.42, 2.00)*		0.828		53	32.5	1.19	*(0.79, 1.79)*		0.411	1.30		*(0.82, 2.08)*		0.266
**Maternal migration characteristics** c																								
Cumulative migration time																								
<3 years *(reference)*		22		19.3	1.00				1.00					57	45.2	1.00				1.00				
≥3 years		9		14.8	0.72	*(0.31, 1.69)*		0.454	0.44	*(0.14, 1.36)*		0.153		35	50.0	1.21	*(0.67, 2.17)*		0.522	1.02		*(0.52, 1.99)*		0.965
Frequency of returning home																								
≥ Once per month *(reference)*		19		16.8	1.00				1.00					63	50.0	1.00				1.00				
< Once per month		10		18.5	1.12	*(0.48, 2.62)*		0.786	1.17	*(0.40, 3.46)*		0.772		25	40.3	0.68	*(0.37, 1.25)*		0.212	0.65		*(0.32, 1.33)*		0.238
Frequency of contact with child																								
≥ Once per month *(reference)*		21		18.3	1.00				1.00					64	48.5	1.00				1.00				
< Once per month		10		19.2	1.07	*(0.46, 2.46)*		0.881	1.38	*(0.50, 3.79)*		0.534		24	43.6	0.82	*(0.44, 1.55)*		0.545	0.85		*(0.40, 1.78)*		0.660

Variables adjusted included school, grade, ethnicity, perceived living standard (as a surrogate of family income) and gender and education of the guardian in the multivariate models.

a Self-efficacy was measured by the smoking self-efficacy questionnaire (SEQ-12), participants with scores below than the 25^th^ percentile of all were defined as low self-efficacy.

b Risks were estimated among all boys.

c Risks were estimated among boys with paternal or maternal migration only.

**Table 3:** Percentages and risk estimations of low self-efficacy^a^ by parental migration among girls.

	n	%	OR_unadj._	*(95% CI)*	P value	OR_adj._	*(95% CI)*	P value	
**Parental migration status** b									
Paternal migration status									
No *(reference)*	132	22.3	1.00			1.00			
Yes, ever	89	28.6	1.39	*(1.02, 1.91)*	0.037	1.36	*(0.95, 1.96)*	0.091	
Yes, currently	31	29.2	1.44	*(0.91, 2.28)*	0.123	1.37	*(0.80, 2.32)*	0.248	
Maternal migration status									
No *(reference)*	192	22.4	1.00			1.00			
Yes, ever	40	47.6	3.15	*(1.99, 4.97)*	<0.001	3.63	*(2.06, 6.39)*	<0.001	
Yes, currently	8	29.6	1.46	*(0.63, 3.38)*	0.380	1.08	*(0.38, 3.08)*	0.891	
Type of parental migration									
No parent migrating *(reference)*	117	21.7	1.00			1.00			
Father migrating only	72	24.3	1.16	*(0.83, 1.63)*	0.380	1.13	*(0.78, 1.63)*	0.536	
Mother migrating only	9	40.9	2.50	*(1.04, 6.00)*	0.040	2.53	*(0.84, 7.66)*	0.099	
Both migrating	35	42.7	2.69	*(1.66, 4.37)*	<0.001	2.88	*(1.62, 5.13)*	<0.001	
**Paternal migration characteristics** c									
Cumulative migration time									
<3 years *(reference)*	68	28.6	1.00			1.00			
≥3 years	51	29.3	1.04	*(0.67, 1.60)*	0.870	1.30	*(0.79, 2.15)*	0.302	
Frequency of returning home									
≥ Once per month *(reference)*	48	31.2	1.00			1.00			
< Once per month	67	26.6	0.80	*(0.52, 1.24)*	0.321	1.06	*(0.63, 1.78)*	0.832	
Frequency of contact with child									
≥ Once per month *(reference)*	71	27.3	1.00			1.00			
< Once per month	40	31.2	1.21	*(0.76, 1.92)*	0.419	1.06	*(0.62, 1.81)*	0.827	
**Maternal migration characteristics** c									
Cumulative migration time									
<3 years *(reference)*	30	45.5	1.00			1.00			
≥3 years	20	41.7	0.86	*(0.40, 1.82)*	0.687	0.86	*(0.29, 2.57)*	0.785	
Frequency of returning home									
≥ Once per month *(reference)*	27	45.8	1.00			1.00			
< Once per month	22	44.0	0.93	*(0.44, 1.99)*	0.854	1.15	*(0.40, 3.27)*	0.801	
Frequency of contact with child									
≥ Once per month *(reference)*	28	40.6	1.00			1.00			
< Once per month	19	54.3	1.74	*(0.77, 3.95)*	0.186	2.87	*(0.89, 9.31)*	0.078	

Variables adjusted included school, grade, ethnicity, perceived living standard (as a surrogate of family income) and gender and education of the guardian in the multivariate models.

a Self-efficacy was measured by the smoking self-efficacy questionnaire (SEQ-12), participants with scores below than the 25^th^ percentile of all were defined as low self-efficacy.

b Risks were estimated among all girls.

c Risks were estimated among girls with paternal or maternal migration only.

Table 4 summarises results of the final model of the hierarchical regression. The type of parental migration was the only migration related variable staying in the model according to the selection criteria of variables. Self-efficacy was a strong predictor of smoking, with the likelihood being higher in boys than in girls. Among boys, only father migrating seemed to be a protective factor against smoking, with the likelihood being even lower and reaching the borderline of significance after entering self-efficacy in the model (OR_adj_ = 0.54, P = 0.050). On the contrary, boys whose mothers only migrated were 3 times more likely to smoke without controlling for self-efficacy (Table 2). However, the risk decreased and became non-significant after adding self-efficacy in the model (OR_adj_ = 1.91, P = 0.140, Table 4). Both parents migrating had no significant effect on smoking without and with consideration of self-efficacy (Tables 2 and 4). Once again, similar situation was also found among girls, though socio-demographic factors were not adjusted for due to few smoking cases.

**Table 4:** Final model of hierarchical regression for past 30-day smoking.

	OR_adj._	*95% CI*	P value	
**Boys**				
Type of parental migration				
No parental migrating (reference)	1.00			
Father migrating only	0.54	*(0.29, 1.00)*	0.050	
Mother migrating only	1.91	*(0.81, 4.51)*	0.140	
Both migrating	0.86	*(0.43,1.73)*	0.671	
Self-efficacy				
Middle+high (reference)	1.00			
Low (<25%)	5.78	*(3.53,* 9.48)	<0.001	
**Girls**				
Type of parental migration				
No parental migrating (reference)	1.00			
Father migrating only	0.87	*(0.16,* 4.79)	0.870	
Mother migrating only	4.50	*(0.46,* 43.66)	0.194	
Both migrating	1.15	*(0.12,* 10.76)	0.900	
Self-efficacy				
Middle+high (reference)	1.00			
Low (<25%)	4.75	*(1.10,* 20.52)	0.037	

Hierarchical logistic regression was performed in boys and girls separately. All parental migration related variables were stepwisely selected using P<0.20 and P<0.25 as entry and removal criteria, whilst only type of parental migration met the criteria and entered in the model. Socio-demographic factors were adjusted among boys only, including school, grade, ethnicity, perceived living standard (as a surrogate of family income) and gender and education of the guardian.

## Discussion

Our study in rural south China found that adolescents with only mothers migrating were more likely to be past 30-day smokers, whilst paternal migration seemed to be protective for adolescent smoking. Both maternal and paternal migration could decrease the level of self-efficacy. Cumulative migration time, frequency of returning home and frequency of contact child during parental migration did not have any extra effect on smoking and self-efficacy. Lower self-efficacy was a strong risk factor for smoking and at least part of the elevated risk of mother only migration only could be mediated by self-efficacy. The effects of parental migration on smoking and self-efficacy were similar between boys and girls.

To our knowledge, it is the first of this kind reporting that maternal migration has an adverse effect on adolescent smoking in rural China, whilst paternal migration tends to be protective. Our results could be explained with consideration of parental smoking in China. Rural male Chinese have a high smoking prevalence (66.0%), whilst female smokers are far less (3.08%) [2]. Although we did not collect data of parental smoking in this study, previous studies have shown that in the study location 71.8%–84.8% of male adults are current smokers, whilst only 1.13%–2.50% of their counterparts currently smoke [19]–[21]. In this context, paternal migration is likely to result in most adolescents left behind free from the influence of their father’s smoking and therefore be protective, as parental smoking has been proved to be a risk factor and mainly attributed to role modelling [22]–[24]. On the other hand, as very few mothers smoke, it is likely that maternal migration leads to adolescents exposed to paternal smoking without a buffering effect of non-smoking mother and therefore increases the risk for offspring smoking [23]. The risk of adolescent smoking increases with the number of smoking parents [23], [24]. A child with one smoking parent is more at risk for smoking than a child with non-smoking parents, and a child with two smoking parents has an increased likelihood to smoke compared to a child with only one smoking parent. In addition, children and adolescents in single-parent families are at elevated risk for smoking, mainly because the modelling effect in single-parent families may differ from that in two-parent families [23]. Specifically, in two-parent families the smoking behaviour from one parent may magnify (if the other parent smokes too) or buffer (if the other parent does not smoke) the behaviour of the other parent. Collectively, our findings of the lower risk of paternal migration and the higher risk of maternal migration is likely to be a result of parental smoking prevalence and its modelling effect on adolescent smoking. In addition, adolescents with parental migration may be at high risk of smoking due to less supervision received and fewer opportunities to communicate with parents [22], [23]. Although we could not distinguish the two pathways in this study, the insignificant effect of both parents migrating at least partly reflects that the latter may be minor compared to the modelling effect.

We hypothesised that cumulative migration time, frequency of returning home and frequency of contact with child during migration would affect the effect of parental migration on adolescent smoking. However, data analyses revealed that these factors might not have significantly extra effects except for parental migration itself. Parental migration in our study was defined as parents who had migrated for at least six months in a row. It is plausible that six months of parental absence may be long enough to affect offspring smoking. The effects of other migration characteristics, if existing, may be minor.

In this study, grandparents were most likely to become the guardian (carer) of adolescents with both parents moving out, followed by uncles/aunts and no adult guardian at all. The majority of adolescents with single migrant parent were cared for by the parent who was at home. No significant relationship was found between the type of guardian and adolescent smoking behaviour, suggesting that extra attentions from their other relatives (if having) may not have significant influence on adolescent smoking (data not shown). The result was in line with our previous study [8]. In addition, there was also no extra intense attention paid to left-behind adolescents from the study schools or the study township before and during the survey.

In this study, both paternal and maternal migration had adverse effects on adolescent self-efficacy, with that of maternal migration being greater. In the final model of hierarchical regression, self-efficacy was apparently the strongest risk factor for smoking (adjusted ORs: 5.78 for boys and 4.75 for girls, Table 4). Furthermore, the decreased risk of father migrating only became even lower and reached the borderline of significance among boys, suggesting that paternal migration may have a direct protective effect on smoking other than through negatively affecting offspring’s self-efficacy. On the contrary, the high likelihood of mother migrating only decreased from 3.03 to 1.91 and the significance vanished, suggesting that self-efficacy may be a mediator of maternal migration on smoking. That is, maternal migration may reduce adolescent self-efficacy and then increase the risk of smoking, implying that it is important and may be effective to reduce the elevated risk of maternal migration through improving adolescent self-efficacy of smoking. Existing interventions for adolescents have provided specific strategies to enhance self-efficacy through reducing barriers of change and developing skills to overcome problems [25], [26]. For example, to identify how to effectively overcome perceived barriers to quitting smoking when adolescent smokers begin to consider quitting; to encourage them to identify and plan ways to overcome the barriers that they are most likely to face when adolescent smokers plan to quit; and to bolster self-efficacy for dealing with new barriers to staying quit and with setbacks that threaten relapse when adolescent smokers quit and are able to stay quit for a while [26].

This study did not find any significant difference in the effects of parental migration on smoking and self-efficacy between boys and girls. However, the effects on female smoking are not conclusive because of the few smoking cases. Further studies should enlarge the sample size to get enough cases to confirm the relationship among girls. In addition, this study must be placed in the context of other limitations. We only studied one rural area in south China and cautiousness is needed before generalising the results to other places. We did not collect the data of parental smoking which weakened our ability to interpret the potential mechanism of parental migration through it. This study focused on family factors. However, some non-family factors (e.g. peer influence) also have influences on adolescent smoking, which may confound the association between parental migration and offspring smoking and therefore should be taken into consideration in future studies. Taking an example of peer smoking, it has been proved to be predictive of initiation, current and ever smoking in adolescents [27]–[29]. In addition, adolescents tend to care a lot about peer opinion, which may affect their perception of themselves and in turn could affect their self-efficacy and smoking. We did not collect such information and it is possible that the impact of parental migration observed in this study may be contingent upon peer influence or other important factors missed in our study. Besides, potential limitations of similar cross-sectional studies also exist in this study. For example, we cannot draw a conclusion on the cause-effect relationship between parental migration and adolescent smoking. All data were reported by the subjects that may introduce recall bias into the study. In addition, the participants may conceal their smoking behaviour because it is prohibited by school rules. We tried to minimize this bias by asking the subjects to complete and submit their questionnaires without any school staff’s presence and approach. Additionally, if the bias exits, it is unlikely to be associated with parental migration. In other words, it is unlikely that the participants with parental migration tended to report smoking more or less compared to those without parental migration. Hence it may not be a major threat to our findings.

In conclusion, our findings suggest that maternal migration increases the risk of adolescent smoking, whilst paternal migration may be protective. Such effects are similar in both genders and tend to be unconditional of the cumulative migration time, frequency of returning home and frequency of contact with child during migration. Self-efficacy is a strong risk factor of smoking. Although both maternal and paternal migration can reduce adolescent self-efficacy of smoking, it seems that self-efficacy is a mediator of the adverse effect of maternal migration, whilst the protective effect of paternal migration is independent of self-efficacy. Our results imply that adolescents whose mothers migrate were more vulnerable to becoming smokers and that improving adolescent self-efficacy may be an effective means to prevent them from smoking.
